# Allergy‐related diseases and early gut fungal and bacterial microbiota abundances in children

**DOI:** 10.1002/clt2.12041

**Published:** 2021-06-28

**Authors:** Kasper Schei, Melanie Rae Simpson, Torbjørn Øien, Saideh Salamati, Knut Rudi, Rønnaug Astri Ødegård

**Affiliations:** ^1^ Department of Clinical and Molecular Medicine Faculty of Medicine and Health Sciences NTNU–Norwegian University of Science and Technology Trondheim Norway; ^2^ Department of Public Health and Nursing Faculty of Medicine and Health Sciences NTNU–Norwegian University of Science and Technology Trondheim Norway; ^3^ Clinic of Laboratory Medicine St. Olav's Hospital Trondheim Norway; ^4^ Regional Centre of Obesity Research and Innovation (ObeCe) St. Olav's Hospital Trondheim Norway; ^5^ Faculty of Chemistry, Biotechnology and Food Science Norwegian University of Life Sciences Ås Norway

**Keywords:** allergy, asthma, children, gut microbiota, mycobiota

## Abstract

**Background:**

The early gut microbiota has been proposed as an important link between environmental exposures and development of allergy‐related diseases. Beyond the widely investigated associations between the gut bacterial microbiota, we investigated the involvement of early gut mycobiota and gut permeability in the pathogenesis of asthma, allergic rhinoconjunctivitis (AR) and eczema.

**Methods:**

In the Probiotics in the Prevention of Allergy among Children in Trondheim trial with maternal probiotic supplementation, we collected faecal samples at four timepoints between 0 and 2 years from a cohort of 278 children. Clinical information on allergy‐related diseases was collected in a paediatric examination at 2 years and questionnaires at 6 weeks and 1, 2 and 6 years. By quantitative PCR and 16S/ITS1 MiSeq rRNA gene sequencing, we analysed the gut bacterial and fungal microbiota abundance and bacterial diversity and explored associations with allergy‐related diseases. We also measured gut permeability markers (lipopolysaccharide‐binding protein [LBP] and fatty acid‐binding protein 2 [FABP2]).

**Results:**

Children with higher fungal abundance at 2 years were more likely to develop asthma and AR by 6 years, odds ratios 1.70 (95% CI: 1.06–2.75) and 1.41 (1.03–1.93), respectively. We explored causal connections, and children with eczema at 1–2 years appeared to have more mature bacterial microbiota, as well as being depleted of *Enterococcus* genus. Although LBP and FABP2 did not correlate with eczema, increased bacterial abundance was associated with increased serum FABP2.

**Conclusions:**

We observed positive associations between gut fungal abundance and allergy‐related disease, but increased gut permeability does not appear to be involved in the underlying mechanisms for this association. Our findings should be confirmed in future microbiota studies.

## 1 BACKGROUND

Allergy‐related diseases, such as asthma, allergic rhinoconjunctivitis (AR) and eczema, are chronic inflammatory diseases. The prevalence of these diseases has increased over the last decades and collectively they are now the most prevalent chronic diseases in childhood.^1^ Environmental factors play an important role in the rising prevalence given the rapidity of its increase, and changing microbial exposures are suspected to be one of the driving factors.^2^ The so‐called *hygiene hypothesis* originally postulated that reduced childhood infections may predispose to allergy‐related diseases; however, this hypothesis has evolved to place a greater emphasis on a depletion or perturbation of the early commensal microbiome and the influence of this disturbance on the developing immune system.^3^, ^4^

Epidemiological studies have identified a number of environmental risk factors for allergy‐related diseases, such as delivery by Caesarean section, formula‐feeding, urban living, less animal contact and Western diet.^5^, ^6^ The influence of each of these factors on allergy‐related disease may in part be due to reduced quantity or diversity of microbial exposures in early life, and through altered development of the gut microbiota in particular. The gut represents a major site where the immune system interacts with vast quantities of microbes. Early low bacterial diversity and varying microbial patterns have been shown to be associated with development of allergy‐related diseases later.^7^, ^8^, ^9^ Whilet the gut microbiota mainly consists of bacteria, fungi also colonise the gut.

The *gut mycobiota* is considered to be a fundamental constituent of the gut microbiota, contributing to around 1% of the microbial cells in the gut.^10^ However, our understanding of the role of mycobiota in allergy‐related disease is scarce, and more knowledge is called for.^11^ The association between gut mycobiota and allergy appears to be more nuanced than for bacteria. Growing up in rural environments with higher fungal content may decrease the risk of allergy‐related diseases,^6^, ^12^ whereas indoor fungal exposure through moulds has been associated with an increased risk.^12^ Two recent small studies have found associations between neonatal fungal dysbiosis and asthma or wheezing in pre‐school age.^8^, ^13^ Furthermore, murine research on fungi has shown that gavage feeding of certain fungi (and possibly perturbing the gut mycobiota) can induce allergic airway disease in mice.^14^

The primary point of contact for both bacterial and fungal members of the gut microbiota and their host is the gut mucosa. The gut mucosal immune system is optimally adapted to tolerate commensal microbes in the gut lumen, while expelling pathogenic invasive microbes by activating proinflammatory reactions, as well as cell wall or intracellular pathogen recognition receptors of epithelial cells.^15^ As such, a more permeable gut mucosa, or *leaky gut*, may allow microbes to penetrate the intestinal epithelial lining more readily and lead to an increased inflammatory response to the gut microbiota. The gut permeability in infancy could be one of the mechanisms by which the gut microbiota is involved in the development of allergy‐related diseases, as higher gut permeability has been observed in both adults and children (5–12 years) with asthma,^16^, ^17^ and eczema.^18^, ^19^ Two such markers of gut permeability are *lipopolysaccharide‐binding protein* (LBP) and *fatty acid‐binding protein 2* (FABP2, also called intestinal‐type FABP, or FABPI). LBP is part of the innate immune response that opsonises lipopolysaccharides (LPS or endotoxin, a cell wall constituent of Gram‐negative bacteria) when this crosses the gut barrier and serves thus as a marker of metabolic endotoxaemia.^20^ FABP2 is expressed in the gut from duodenum to caecum and is a marker of gut permeability and enterocyte turnover or damage.^21^ Higher circulating levels of both markers are observed in neonatal necrotising enterocolitis.^22^

Several findings indicate that the gut microbes are associated with allergy development. However, the potential association between early gut fungal and bacterial microbiota, and development of allergy‐related diseases is largely unknown. The aim of this study was, therefore, to explore the associations between the early gut fungal and bacterial microbiota and gut permeability during the first 2 years of life, and the development of allergy‐related diseases up to 6 years of age in a prospective cohort.

## 2 METHODS

### 2.1 Materials

The faecal samples in this study were collected during the Probiotics in the Prevention of Allergy among Children in Trondheim (ProPACT) study, a Norwegian randomised placebo‐controlled trial investigating the use of maternal probiotic supplementation in the prevention of eczema and other allergy‐related diseases.^23^ Briefly, mothers were randomised to drink either sterile placebo milk or probiotic milk containing three probiotic bacteria (5 × 10^10^ colony‐forming units [CFUs] of both *Bifidobacterium animalis* subsp. *lactis* Bb‐12 and *Lactobacillus rhamnosus* GG [LGG], and 5 × 10^9^ CFUs of *Lactobacillus acidophilus* La‐5) from 36 weeks to 3 months postpartum. The children whose mothers had received probiotics had a 39% reduction in the chance of having eczema by 2 years of age.^24^

#### 2.1.1 Faecal samples

**FIGURE 1:**
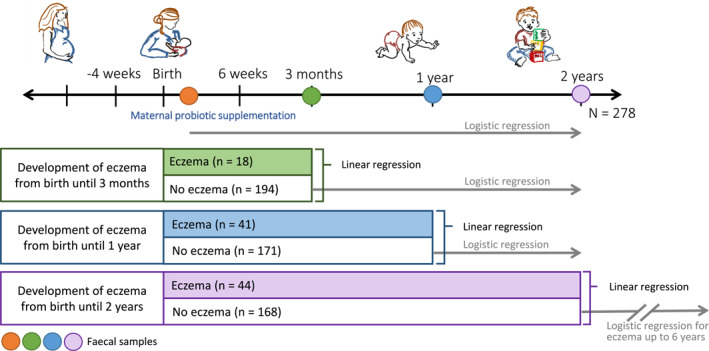
Causality analysis. Overview of how the causality analysis between fungal and bacterial abundances and eczema was performed. Linear regressions were used to estimate if eczema diagnosed at or prior to each timepoint predicted fungal or bacterial abundance at that timepoint. Logistic regressions were used to estimate if fungal or bacterial abundance at each timepoint predicted eczema up to 2 years (6 years for the lowest analysis) among those with no history of eczema by that timepoint. The included participants in this analysis were those who could develop eczema at 0–6 years of age. This number adds up to *n* = 212, as three participants did not provide the presentation age of eczema in the questionnaires

Out of 415 included children, the 278 children who attended the clinical follow‐up at 2 years were included in the current study (Table 1, Figure 1). The mothers included in the current analysis were 8 months older and of longer education and their children had received more antibiotics between 1 and 2 years of age; other than that the groups were comparable.^25^ These children provided in total 1015 faecal samples at up to four timepoints (10 days, 3 months, 1 and 2 years). The parents collected the samples from the diaper into a Cary‐Blair transport medium (about 20 times dilution) with an enclosed spoon, and they were asked to place the samples in a −18°C freezer immediately before transport to the laboratory and storage at −80°C.

**TABLE 1:** Participant characteristics (*N* = 278)

Participant characteristics		*n* ^a^
Girls, *n* (%)	149 (53.6)	278
Caesarean section, *n* (%)	35 (12.8)	273
Birth weight, *g*, mean (SD)	3633 (486)	278
Birth length, cm, mean (SD)	50.5 (1.9)	242
Gestational age at birth, weeks, mean (SD)	40.3 (1.6)	274
Months of breastfeeding, months, mean (SD)	11.0 (4.6)	260
Antibiotics administration, *n* (%)		
‐ Within 6 weeks	6 (2.5)	241
‐ Within 1 year	35 (13.6)	258
‐ Within 2 years	166 (41.7)	278
Glucocorticoid inhalations before 2 years, *n* (%)	4 (1.4)	278
Eczema within 6 years, *n* (%)	53 (24.7)	215
Allergic rhinoconjunctivitis within 6 years, *n* (%)	22 (10.6)	207
Asthma within 6 years, *n* (%)	10 (4.0)	221

Abbreviation: SD, standard deviation.

^a^ Number of observations included in the analysis.

#### 2.1.2 Follow‐ups and definitions of allergy‐related diseases

The parents were asked to complete questionnaires on home environment of the child at 6 weeks after birth, 1 year and 2 years, and child health questionnaires at 1, 2 and 6 years. Additionally, a clinical evaluation of allergy‐related diseases was performed by a paediatrician at 2 years of age and by research assistants at 6 years of age. Current eczema (within the last year) at 2 or 6 years was defined according to The U.K. Working Party's diagnostic criteria for atopic eczema.^26^, ^27^ Ever eczema at 2 or 6 years was defined using the questionnaire responses if the parents reported that the child had ‘ever had eczema’ and ‘ever had a recurring itchy rash during 6 months’. Similarly, asthma and AR were defined using the questionnaire if the parents reported that the child was ‘ever diagnosed with asthma by a physician’ for asthma and ‘ever had hay‐fever, allergic rhinitis or allergic conjunctivitis’ for AR.

### 2.2 Methods

#### 2.2.1 Microbiota analyses

The microbiota analyses were fully explained in a previous article.^25^ The faecal samples were homogenised before DNA extraction by a bacterial protocol,^28^ since there were no validated protocols at the time for fungal DNA extraction. The microbial abundances were quantified by quantitative PCR with bacteria‐targeted primers (V3‐V4 part of the 16S rRNA gene),^29^ and fungi‐targeted primers (ITS1 part of 18S rRNA gene).^30^ Out of the 1015 samples, 999 (98%) and 653 (64%) had quantifiable levels of bacteria and fungi, respectively (Supplementary Table S1A).^25^ The ITS1 qPCR cut‐off value was set to the negative control if fungal abundance was lower than negative control. For samples without quantifiable levels of fungus (CT [cycle threshold] ≥ 45, 260 samples), the abundance per ml was set to half of the negative control for the purposes of analyses. A further 102 fungal samples could not be analysed due to technical issues and were thus excluded (Supplementary Table S1B). No differences were detected in mode of delivery, weaning age, antibiotic administration, number of pets or siblings between these three groups (detected, non‐detected and technical issues), nor in allergy‐related diseases. Those with non‐detected fungal abundance at 1 year received solid foods on average half a month before those with detected fungal abundance at 1 year, yet both these groups were included in the analysis. This was adjusted for in the main analysis, and there were minimal changes in the effect estimates so crude estimates were used. Recently, the quantification of the rRNA 18S/ITS1 gene region has been established as a good marker of fungal abundance estimation.^31^, ^32^ Subsequently, we used Illumina MiSeq for sequencing and the bioinformatic processes were done in Quantitative Insights Into Microbial Ecology pipeline with UPARSE for operational taxonomic unit clustering.^28^, ^33^ Rarefaction cut‐offs were 2000 reads/sample for bacteria and 6000 reads/sample for fungi according to previous studies.^28^, ^33^ Bacterial sequencing was performed on 757 samples (75%). Samples with fungal ITS1 qPCR CT values > 35 were not sequenced due to low total quantities, and those with less than 6000 reads per sample were excluded after sequencing due to insufficient data. This resulted in that 37 (4%) samples were sequenced for fungi. For this reason, only bacterial diversity is used in the analysis. For annotation, Greengenes Database was used for bacteria, and a self‐curated concordance system was used for fungi as there were no well‐established methods for fungal annotation.^33^

#### 2.2.2 Gut permeability markers

We analysed serum levels of LBP and FABP2. LBP was measured by ELISA (Human LBP, Merck, own positive controls) and FABP2 by ELISA (Human FABP2/I‐FABP Quantikine ELISA Kit, R&D Systems, producer's positive controls), following the producers' protocols, including negative controls on all plates.

### 2.3 Statistics

The data from both the probiotic and placebo arm of the ProPACT trial are used in the current analysis. Previous analysis of the infant stool samples has indicated that only LGG appears to be transferred from mother to child. Although we observed greater abundance and presence of LGG at 10 days and 3 months in infant stool samples from the probiotics group, the relative abundance of LGG was still low and did not persist at 1 or 2 years of age.^23^ Furthermore, maternal probiotic supplementation was not associated with any change in the overall microbial composition of diversity at any age,^23^ or with fungal abundance.^33^ Since the overall microbial composition of the stool samples was unaffected by the maternal supplementation, we have pooled the probiotic and placebo arms of the ProPACT trial.

Firstly, we investigated statistical associations between the development of the allergy‐related diseases by 6 years of age and the absolute abundance of bacteria and fungi in the stool samples using logistic regression models. Log‐transformed bacterial and fungal concentrations at all four faecal sampling points were analysed against ever allergy‐related diseases up to 6 years.

Whilst these models identify associations between the microbial abundances and allergy‐related disease, they do not provide insight into whether the higher bacterial or fungal abundances influence allergy‐related disease development, or if allergy‐related disease development has an impact on the abundance of bacteria or fungi. For this reason, we considered each of these scenarios by exploring subgroups of the children based on their age of onset of eczema and stool samples collection (Figure 1). Specifically, to determine if higher microbial abundances in early life influenced allergy‐related disease development, we used logistic regression models to estimate the risk of developing eczema by 2 years of age associated with microbial abundance in the subgroup of infants who had not yet developed eczema when the 10‐day, 3‐month and 1‐year stool sample was collected, respectively. Finally, we estimated the risk of developing eczema by 6 years of age based on fungal abundance at 2 years of age among children who had not developed eczema by 2 years of age. In order to investigate if allergy‐related disease development had an impact on the abundance of bacteria or fungi, we used linear regression models to estimate the change in microbial abundance based on whether or not the child had developed eczema before the time of stool collection. These exploratory analyses of causality were limited to eczema as this was the most prevalent. A separate regression model was assessed for each collection timepoint.

Statistical calculations were performed in StataMP16 and RStudio was used for microbiome analysis. Microbial ordination was performed in R with the *phyloseq* package,^34^ and specific microbial taxa were analysed with Linear Discriminant Analysis Effect Size (LEfSe)^35^ in the on‐line Galaxy module with a threshold Linear Discriminant Analysis score of 2.0, in which a correction for multiple testing is included. ANCOM‐BC was used for longitudinal taxonomic statistic.^36^ Estimated associations are presented with 95% confidence intervals.

## 3 RESULTS

### 3.1 Fungal and bacterial abundance and allergy‐related diseases

**FIGURE 2:**
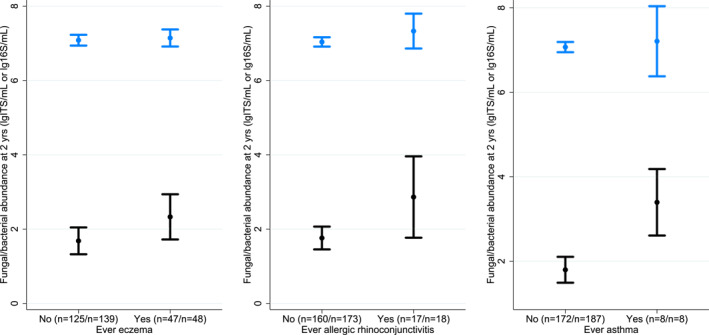
Fungal and bacterial abundance and allergy‐related diseases. These figures depict the mean and 95% CI of fungal (black) and bacterial (blue) abundance at 2 years of age for children with ever allergy‐related disease at 0–6 years, supplementing the logistics regression analyses. The numbers within brackets represent the number of participants in each group for fungal and bacterial analysis, respectively

We found positive associations between the cumulative incidence of ever asthma and ever AR at 6 years and higher faecal fungal abundance at 2 years, with odds ratios (ORs) of 1.70 (95% CI [confidence interval]: 1.06–2.75) and 1.41 (1.03–1.93), respectively (Figure 2, Supplementary Figures S1 and S2, Supplementary Tables S2 and S3). We also found a positive association between eczema up to 6 years and fungal abundance at 2 years (OR 1.18, 95% CI: 0.99–1.40), although the confidence interval for this estimate was wide, and we cannot exclude that there is no association. Effect estimates only varied minimally when corrected for mode of delivery, weaning age, introduction of solid foods, antibiotic administration or the number of pets and siblings (Supplementary Table S4).

We investigated the possible directions of causality for eczema by analysing whether fungal abundance was present before debut of allergic disease, or if allergic disease appeared before the increased fungal abundance (Figure 1). Considering first the subgroups of children who had not developed eczema by the time the respective stool samples were collected, we found no conclusive associations between fungal abundance at 10 days, 3 months or 1 year and the later development of eczema (Supplementary Figure S3 and Supplementary Tables S5 and S6). Nevertheless, fungal abundance at 2 years was positively associated with the development of eczema between 2 and 6 years of age (OR: 1.82, 95% CI: 0.97–3.44). Whilst this observation would not be considered statistically significant, it provides some support for the idea that fungal abundance may increase before eczema development.

On the other hand, when we considered the association between an existing eczema diagnosis and fungal abundance at each timepoint, we found that eczema debut before 3 months, 1 or 2 years was not conclusively associated with fungal abundance at these timepoints (Supplementary Figure S4 and Supplementary Table S5). Regarding bacterial abundance and allergy‐related diseases, we found no clear associations in equivalent analyses (Supplementary Table S5).

### 3.2 Microbial taxa and eczema

**FIGURE 3:**
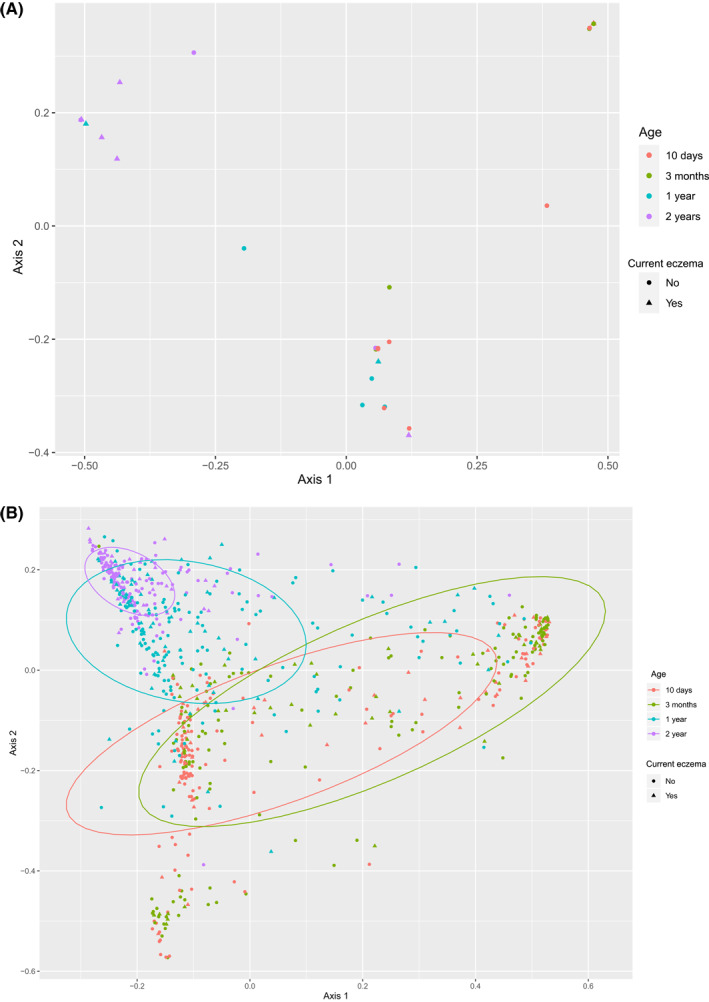
Principal Coordinate Analysis (PCoA) of fungi and bacteria. PCoA is a multivariate ordination method that depicts the all‐over taxonomic variation between samples, in which two samples with similar taxa and abundances are put closer and dissimilar samples would be placed far apart. The 2‐years samples cluster top‐left for both fungi and bacteria, whereas neonatal samples are more wide‐spread. (A) describes fungal taxa distribution. (B) describes bacterial taxa distribution. Ellipses contain 70% of each age group

Principal coordinate analysis (PCoA) ordination plots indicated that the fungal and bacterial variance of the microbiota is partially determined by age (Figure 3A,B and Supplementary Figure S5). The fungal PCoA plot showed that the majority of 2‐year samples with current eczema clustered to the upper‐left of the plot; statistical testing was not feasible due to few samples. Whilst there was no clear separation of samples in the bacterial PCoA plot based on eczema development, we observed a general trend that 1‐ and 2‐year samples from children with current eczema at 2 years were over‐represented the upper‐left section of the PCoA plot. To investigate this more objectively, we identified the spread samples for each age category by constructing age‐specific ellipses which encompassed 70% of microbiota samples from that age group (Figure 3). A greater proportion of the microbiota samples from 2‐year‐old children with current eczema at 2 years clustered within the 2‐year ellipse, indicating a less infantile bacterial microbiota in these children (Risk Ratio [RR]: 1.84, 95% CI: 1.02–3.34) (Figure 3, Supplementary Table S7). Furthermore, the microbiota samples from 1‐year‐old children with current eczema at 2 years were over‐represented within the 2‐year ellipse (RR: 1.28, 95% CI: 1.03–1.60, Figure 3). This suggests that a less infantile bacterial microbiota already at 1 year could indicate an increased chance of eczema at 2 years of age. Possible explanatory variables like antibiotic administration, mode of delivery, breastfeeding and gestational age did not seem to explain the variance of the PCoA plot (Supplementary Figure S6A–D).

**FIGURE 4:**
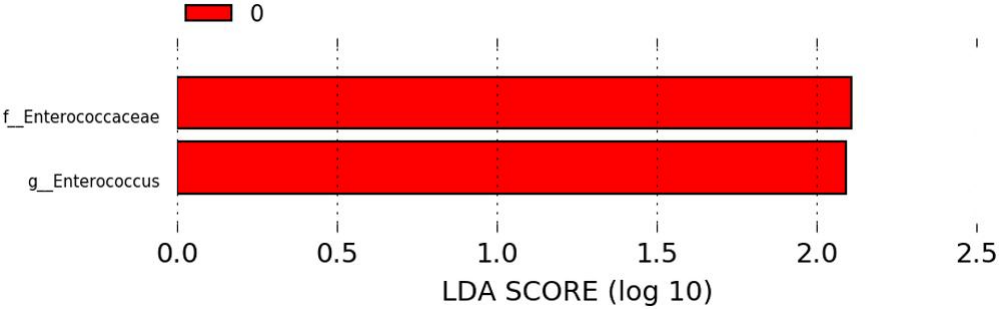
Linear discriminant analysis effect size plot. *Enterococcus* sp. and Enterococcaceae were significantly over‐represented in healthy children at 2 years (0, red) compared to children with eczema

A LEfSe analysis of the fungal and bacterial microbiota was performed for different age groups to explore if specific taxa correlated with fungal abundance and eczema. Specific taxa were found significantly over‐ and underrepresented in children with and without ever eczema (Figure 4), and in children with high and low fungal abundance (Figure 4). In particular, we found high abundances of *Enterococcus* sp. at 2 years in children without eczema. Several taxa were also under‐ and over‐represented with age and fungal abundance (Supplementary Figure S7A,B). Longitudinal ANCOM‐BC provided no statistically significant associations with allergy‐related diseases.

### 3.3 Fungal and bacterial abundance, markers of intestinal permeability and development of eczema

To evaluate if the associations between microbial abundance and allergy development were explained through gut permeability, the two gut permeability markers FABP2 and LBP were measured in serum at 10 days, 3 months, 1 and 2 years. There was lower risk of ever eczema for increasing FABP2 at 1 year (OR: 0.49, 95% CI: 0.24–1.00, logistic regression, Supplementary Table S8). Increased bacterial abundance showed increased FABP2 (*β* 0.05, 95% CI: 0.00–0.11, log [pg/ml]/log [16S/ml], mixed linear regression); there were however no statistically significant associations between fungal abundance or bacterial diversity and FABP2.

There were neither statistically significant associations between LBP and eczema, nor between LBP and fungal abundance (Supplementary Table S8). Increased bacterial diversity was associated with higher LBP at 3 months (*β* 0.32, 95% CI: 0.05–0.59), but with lower LBP at 2 years (*β* −0.22, 95% CI −0.43 to −0.01).

## 4 DISCUSSION

In this population cohort following children from birth to 6 years, we found that children with higher fungal abundance at 2 years of age were more likely to have allergy‐related diseases within the first 6 years of life. In an attempt to identify the causal direction of this associations, subgroup analyses suggested that higher fungal abundance at 2 years may precede later eczema development. However, this should be interpreted cautiously because of the uncertainty around this estimate. We also observed that early bacterial microbiota succession might appear prior to eczema, however markers of intestinal permeability were not consistently associated with eczema, fungal or bacterial abundance, or bacterial diversity.

We found that fungal abundance was associated with allergy‐related diseases. This is in line with a small case‐control study with 20 Ecuadorian neonates,^13^ where higher fungal abundance was associated with allergy‐related diseases. Although the analysis between fungal abundance and allergy‐related diseases was not constructed to prove causality, our causality analysis could imply that fungal abundance was present prior to development of allergy‐related diseases. Fungi are eukaryotes that are evolutionarily closer to humans than bacteria, sharing a great number of common biochemical pathways. Thus, they could impact human pathways through production of human‐like substances. Fungi are well‐known producers of prostaglandins and leukotrienes.^37^ The *Candida* yeast, and likely other fungi, can produce prostaglandins that may reduce gut macrophage activity against fungi and thereby improve fungal gut colonisation.^38^ By orchestrating the immune system through self‐produced priming cytokines, fungi could in theory induce the native T helper (T_H_) cells to pursue a T_H_2 direction, which could create both a fungus‐friendly gut environment and a predisposition to allergy‐related disease development.^39^ Whilst this is a plausible biological explanation for a causal association between gut mycobiota and later allergy‐related diseases, our results cannot exclude that the association could also be driven by the mechanisms working in the opposite direction. Allergy‐related diseases and their underlying immunopathology could themselves promote a fungus‐friendly gut environment.

Our taxonomic analyses suggested that 1‐year‐olds were prone to develop eczema if their bacterial microbiota compositions more closely resembled the composition seen in 2‐year‐olds. Neither delivery, antibiotics administration nor breastfeeding appeared to explain the taxonomic succession from 1 to 2 years. The absence of the lactic‐acid bacteria *Enterococcus* spp. at 2 years was associated with eczema at 2 years. *Enterococcus* depletion has also been found in allergic 8‐year‐old Swedish children.^40^ Some enterococci are also used as probiotics.^41^ *Enterococcus faecalis* has been found to reduce T_H_17 stimulation and ameliorate allergic airways disease in mice,^4^ which is consistent with our finding that *Enterococcus* sp. is overabundant among infants who do not develop eczema. *Enterococcus* is also a partial fungal antagonist by decreasing the virulence and hyphal morphogenesis of *Candida*.^42^ Depletion of important gut microbes that balance the microbiota and prevent fungal overgrowth, may therefore play a role in eczema and development of other allergy‐related diseases. As only few samples were sequenced for fungi, the fungal LEfSe analysis should be interpreted with care.

We found that FABP2 increased with bacterial abundance, whereas LBP was not associated to neither fungal nor bacterial abundance. As none of these markers were associated with eczema, the connection between fungal abundance and eczema does probably not involve increased gut permeability and metabolic endotoxaemia. However, the finding could indicate that bacterial abundance might affect the gut wall permeability. Different methods and younger participants in our study could explain why we could not replicate the gut permeability–allergy association found in older and smaller studies.

### 4.1 Limitations

Whilst fungal abundance was quantifiable for approximately two thirds of the stool samples, only a minority of these could be sequenced and we therefore have limited opportunity to investigate the role of the mycobiota composition on allergy‐related disease. Also, the number of repeats of the fungal ITS gene sequence varies between fungal species, which could increase the random error in the analysis. A fungal DNA extraction technique would be preferred if such were available, to improve quantification and sequencing.^32^

### 4.2 Strengths

In this prospective cohort study of children from a general population followed for 6 years, we pursued an explorative approach with the intention to reveal mechanisms underlying the associations between both the fungal and bacterial microbiota and allergy‐related diseases. A large sample size and up to four consecutive samples at different ages were analysed. Furthermore, we have quantified the total fungal and bacterial abundance and included markers of gut permeability to explore potential causal pathways of these findings.

## 5 CONCLUSIONS

To conclude, our study showed that gut fungal abundance at 2 years was associated with the development of allergy‐related diseases in the first 6 years of life. Whilst we could not conclusively determine the causal direction of this association, this study indicated that the fungal and bacterial microbiota may both contribute to the complex pathogenesis of allergy‐related disease. To approach a pathophysiologic mechanism, further scientific efforts should be put into larger infancy cohorts with even more frequent early measurements and improved methods of gut fungal and bacterial microbiota and allergy‐related diseases.

## CONFLICT OF INTEREST

The authors declare that the research was conducted in the absence of any commercial or financial relationships that could be construed as a potential conflict of interest.

## AUTHORS’ CONTRIBUTIONS

Kasper Schei did the microbiome data generation, the statistical analysis, interpretation and drafted the initial manuscript; Melanie Rae Simpson contributed to the concept, statistical analysis and interpretation and reviewed and revised the manuscript; Knut Rudi was involved in the microbiome data generation and reviewed and revised the manuscript; Torbjørn Øien designed the study, enrolled the participants, coordinated and supervised the data collection and reviewed and revised the manuscript; SS aided in the conceptualisation and design of the study and reviewed and revised the manuscript; Rønnaug Astri Ødegård supervised the study, conceptualised and designed the study, interpreted the data and reviewed and revised the manuscript. All authors approved of the final manuscript as submitted and agree to be accountable for all aspects of the work.

## Supporting information

Supplementary Material 1
