# Supplementary material for: Allergy‐related diseases and early gut fungal and bacterial microbiota abundances in children
Source: Clin Transl Allergy. 2021 Jun 28;11(5):e12041. doi: 10.1002/clt2.12041 (PMC8238386; doi:10.1002/clt2.12041)
Supplement: Supplementary file 1 — Supplementary Material 1 [file CLT2-11-e12041-s001.docx]

Supporting information

**SUPPLEMENTARY TEXT**

*Statistical associations between fungal abundance, bacterial abundance and bacterial alpha diversity.* We did not observe any convincing associations between fungal abundance at 10 days, 3 months or 1 year and allergy-related diseases up to 6 years of age. Nor did we find any certain associations between faecal bacterial abundance or bacterial alpha diversity at any of the time-points for stool collection and ever allergy-related disease (asthma, AR or eczema) from 0-6 years.

**SUPPLEMENTARY STATISTICAL TABLES**

**Supplementary Table 1A: rRNA gene quantification and 16S/ITS rRNA gene region sequencing of faecal samples**

|  | 10 days | 3 months | 1 year | 2 year | Total |
| --- | --- | --- | --- | --- | --- |
| All faecal samples (count) | 274 | 246 | 247 | 248 | 1015 |
| Detected bacterial DNA (16S rRNA gene region) | 266 (97 %) | 243 (99 %) | 247 (100 %) | 243 (98 %) | 999 (98 %) |
| Sequenced 16S rRNA V3-V4 gene region amplicons (after rarefaction) ^a^ | 178 (65 %) | 193 (78 %) | 216 (87 %) | 170 (69 %) | 757 (75 %) |
| Detected fungal DNA (ITS rRNA gene region) | 153 (54 %) | 148 (60 %) | 163 (66 %) | 189 (76 %) | 653 (64 %) |
| Sequenced ITS gene region amplicons (after rarefaction)^a^ | 15 (6 %) | 4 (2 %) | 7 (3 %) | 11 (4 %) | 37 (4 %) |

^a^ Samples were sequenced if the qPCR cycle threshold was < 35 cycles to provide trustworthy results in the sequencing procedure. Few samples were excluded due to rarefaction.

This table is copied from a previous article ^1^ with permission.

**Supplementary Table 1B: Number of faecal samples from children according to the detectability of fungal abundances in faecal samples**

|  | **10 days** | **3 months** | **1 year** | **2 years** | **Total** |
| --- | --- | --- | --- | --- | --- |
| Samples with technical issues | 31 | 28 | 24 | 19 | 102 (10 %) |
| Samples with non-detected fungal abundance | 90 | 70 | 60 | 40 | 260 (26 %) |
| Samples with detected fungal abundance | 153 | 148 | 163 | 189 | 653 (64 %) |
| Total number of faecal samples | 274 | 246 | 247 | 248 | 1015 (100 %) |

**Supplementary Table 2: Odds ratio for the development of allergy-related disease by 6 years of age associated with fungal and bacterial abundance at 2 years of age**

|  | **Fungal abundance** | | **Bacterial abundance** | |
| --- | --- | --- | --- | --- |
|  | **ORs, 95 % CI** | **n** | **OR, 95 % CI** | **n** |
| Ever asthma at 6 years | 1.70, 1.06 to 2.75 | 180 | 1.21, 0.53 to 2.78 | 195 |
| Ever allergic rhinoconjunctivitis at 6 years | 1.41, 1.03 to 1.93 | 177 | 1.49, 0.85 to 2.62 | 191 |
| Ever eczema at 6 years | 1.18, 0.99 to 1.40 | 172 | 1.08, 0.74 to 1.60 | 187 |

Logistic regressions with allergy-related disease as dependent variable and fungal abundance as independent variable. ORs – odds ratios, CI – confidence intervals, n – number of observations in analysis

**Supplementary Table 3: Odds ratio for allergy-related diseases at 6 years associated with fungal abundance at 2 years of age.**

|  | **2 years** |
| --- | --- |
| **Fungal detection against ever eczema (OR, 95 % CI, n)** | 1.36, 0.54 to 3.40, 172 |
| **Fungal detection against ever allergic rhinoconjunctivitis (OR, 95 % CI, n)** | 1.59, 0.34 to 7.35, 177 |
| **Fungal detection against ever asthma (OR, 95 % CI, n)** | ^1^, 180 |

*Detected/no detection for the main outcomes*

^1^ Not possible to calculate as no participants without detectable fungi (n=31) had ever asthma.

Logistic regressions for fungal detection against allergy-related diseases. β – beta coefficient, CI – Confidence intervals, n – number of observations in analysis, ORs – Odds ratios

| **OR,  95 % CI,  n, p** | **Crude** | **Adjusted for characteristics** | | | | | |
| --- | --- | --- | --- | --- | --- | --- | --- |
|  |  | **Mode of delivery** | **Age of weaning (months)** | **Antibiotics within 2 years** | **Introduction of solid foods (months)** | **Pets at 2 years** | **Siblings at 2 years** |
| Ever eczema at 6 years | OR 1.18, 0.99-1.40, n=172, p=0.07 | OR 1.15, 0.96-1.38, n=168, p=0.12 | OR 1.20, 0.99-1.45, n=160, p=0.06 | OR 1.19, 0.99-1.42, n=171, p=0.06 | OR 1.14, 0.94-1.39, n=124, p=0.17 | OR 1.16, 0.97-1.40, n=160, p=0.11 | OR 1.23, 0.95-1.60, n=102, p=0.11 |
| Ever AR at 6 years | OR 1.41, 1.03-1.93, n=177, p=0.03 | OR 1.43, 1.03-2.00, n=173, p=0.03 | OR 1.40, 0.02-1.92, n=164, p=0.04 | OR 1.40, 1.02-1.92, n=176, p=0.04 | OR 1.27, 0.92-1.78, n=127, p=0.14 | OR 1.36, 1.00-1.86, n=167, p=0.05 | OR 1.28, 0.83-1.94, n=92, p=0.25 |
| Ever asthma at 6 years | OR 1.70, 1.06-2.75, n=180, p=0.03 | OR 1.74, 1.06-2.86, n=176, p=0.03 | OR 1.74, 1.04-2.90, n=167, p=0.04 | OR 1.74, 1.06-2.85, n=179, p=0.03 | OR 1.56, 0.90-2.70, n=130, p=0.12 | OR 1.66, 1.02-2.69, n=132, p=0.04 | OR 1.83, 0.95-3.55, n=106, p=0.07 |

**Supplementary Table 4: Crude and corrected effect estimates of logistic regression between fungal abundance at 2 years and allergy-related diseases.**

AR – allergic rhinoconjunctivitis; CI – confidence intervals, OR – odds ratio.

**Supplementary Table 5: Causality analysis for development of eczema within 6 years and fungal and bacterial abundance.**

|  | **Fungal abundance** | | **Bacterial abundance** | |
| --- | --- | --- | --- | --- |
|  | **ORs, 95 % CI** | **n** | **ORs, 95 % CI** | **n** |
| Abundance (10 days) against eczema incidence (10 days-2 years) | 0.98, 0.86 to 1.12 | 179 | 1.10, 0.75 to 1.61 | 199 |
| Abundance (3 months) against eczema incidence (3 months-2 years) | 0.96, 0.80 to 1.15 | 153 | 1.13, (0.72 to 1.79) | 174 |
| Abundance (1 year) against eczema incidence (1-2 years) | 0.83, 0.56 to 1.23 | 142 | 1.05, 0.56 to 1.96 | 156 |
| Abundance (2 years) against eczema incidence (2-6 years) | 1.82, 0.97 to 3.44 | 130 | 1.43, 0.46 to 4.38 | 143 |
|  | **β, 95 % CI** |  | **β, 95 % CI** |  |
| Eczema incidence (≤ 3 months) against abundance (3 months) | -0.16, -1.42 to 1.09 | 161 | -0.02, -0.45 to 0.43 | 182 |
| Eczema incidence (≤ 1 year) against abundance (1 year) | 0.08, -0.78 to 0.94 | 170 | -0.08 (-0.50 to 0.34) | 190 |
| Eczema incidence (≤ 2 years) against abundance (2 years) | 0.33, -0.42 to 1.08 | 168 | 0.05 (-0.25 to 0.35) | 183 |

Logistic and linear regressions for causality analysis. β – beta coefficient, CI – Confidence intervals, n – number of observations in analysis, ORs – Odds ratios

**Supplementary Table 6: Causality analysis for development of eczema within 6 years and fungal and bacterial detection.**

|  | **Fungal abundance** | |
| --- | --- | --- |
|  | **ORs, 95 % CI** | **n** |
| Fungal detection (10 days) against eczema incidence (10 days-2 years) | 0.69, 0.33 to 1.42 | 180 |
| Fungal detection (3 months) against eczema incidence (3 months-2 years) | 0.84, 0.37 to 1.90 | 153 |
| Fungal detection (1 year) against eczema incidence (1-2 years) | 0.64, 0.11 to 3.66 | 142 |
| Fungal detection (2 years) against eczema incidence (2-6 years) | ^1^ | 130 |
|  | **OR, 95 % CI** |  |
| Eczema incidence (≤ 3 months) against fungal detection (3 months) | 1.08, 0.34 to 3.38 | 161 |
| Eczema incidence (≤ 1 year) against fungal detection (1 year) | 0.72, 0.31 to 1.67 | 170 |
| Eczema incidence (≤ 2 years) against fungal detection (2 years) | 1.00, 0.39 to 2.55 | 168 |

^1^ Not possible to calculate as none of participants without detectable fungi (n = 24) developed eczema between 2 and 6 years, whereas 5 out of the 106 patients with detectable fungi developed eczema between 2 and 6 years.

Logistic and linear regressions for fungal detection against allergy-related diseases. β – beta coefficient, CI – Confidence intervals, n – number of observations in analysis, ORs – Odds ratios

**Supplementary Table 7: Risk ratio for the development of eczema at 2 years associated with the maturity of bacterial gut microbiota at 1 and 2 years of age**

| **Independent variable against dependent variable** | **RR, 95 % CI** | **n** |
| --- | --- | --- |
| Mature bacterial gut microbiota at 2 years against current eczema at 2 years | 1.84, 1.02 to 3.34 | 169 |
| Mature bacterial gut microbiota at 1 year against current eczema at 2 years | 1.28, 1.03 to 1.60 | 180 |

Chi-square test for gut maturity. RR – risk ratio.

**Supplementary Table 8: Gut permeability analysis**

| **Independent variable against dependent variable** | **10 days** | **3 months** | **1 year** | | **2 years** |
| --- | --- | --- | --- | --- | --- |
| **FABP2 against ever eczema (OR, 95 % CI, n)** | 0.64, 0.13 to 3.16, 34 | 1.18, 0.39 to 3.51, 43 | 0.49, 0.24 to 1.00, 100 | | 1.48, 0.65 to 3.37, 90 |
| **FABP2 against fungal abundance (β, 95 % CI, obs.)** | Linear mixed model: 0.00, -0.02 to 0.02, 518 | | | | |
|  | 0.07, -0.02 to 0.16, 58 | -0.11, -0.24 to 0.02, 58 | | 0.05, -0.03 to 0.14, 123 | -0.02, -0.10 to 0.06, 148 |
| **FABP2 against bacterial abundance (β, 95 % CI, obs.)** | Linear mixed model: 0.05, 0.00 to 0.11, 569 | | | | |
|  | 0.02, -0.12 to 0.17, 90 | 0.14, 0.01 to 0.27, 100 | | 0.05, -0.04 to 0.13, 190 | 0.01, -0.09 to 0.11, 189 |
| **FABP2 against bacterial diversity (β, 95 % CI, obs.)** | Linear mixed regression: -0.04, -0.14 to 0.07, 437 | | | | |
|  | -0.05, -0.32 to 0.23, 60 | 0.10, -0.10 to 0.31, 72 | | -0.06, -0.25 to 0.12, 167 | -0.09, -0.29 to 0.11, 138 |
| **LBP against ever eczema (OR, 95 % CI, n)** | 1.28, 0.53 to 3.10, 77 | 0.74, 0.38 to 1.42, 86 | | 0.73, 0.46 to 1.20, 167 | 0.86, 0.48 to 1.53, 169 |
| **LBP against fungal abundance (β, 95 % CI, obs.)** | Linear mixed model: 0.01, -0.02 to 0.04, 520 | | | | |
|  | 0.01, -0.04 to 0.06, 83 | 0.01, -0.07 to 0.09, 89 | | 0.01, -0.04 to 0.06, 175 | 0.00, -0.05 to 0.05, 173 |
| **LBP against bacterial abundance (β, 95 % CI, obs.)** | Linear mixed model: 0.03, -0.03 to 0.09, 571 | | | | |
|  | 0.01, -0.13 to 0.14, 90 | -0.02, -0.18 to 0.15, 101 | | 0.05, -0.05 to 0.15, 191 | 0.02, -0.09 to 0.13, 189 |
| **LBP against bacterial diversity (β, 95 % CI, obs.)** | Linear mixed regression: -0.03, -0.14 to 0.09, 437 | | | | |
|  | -0.06, -0.36 to 0.23, 60 | 0.32, 0.05 to 0.59, 73 | | -0.11, -0.32 to 0.11, 167 | -0.22, 0.43 to -0.01, 137 |

Analysis of gut permeability markers fatty acid-binding protein 2 (FABP2) and lipopolysaccharide-binding protein (LBP), mixed linear regressions from 10 days to 2 years, and linear regressions or logistic regression for each age. β – beta coefficient, CI – confidence intervals, OR – odds ratio.

**SUPPLEMENTARY FIGURES**

**A**

**
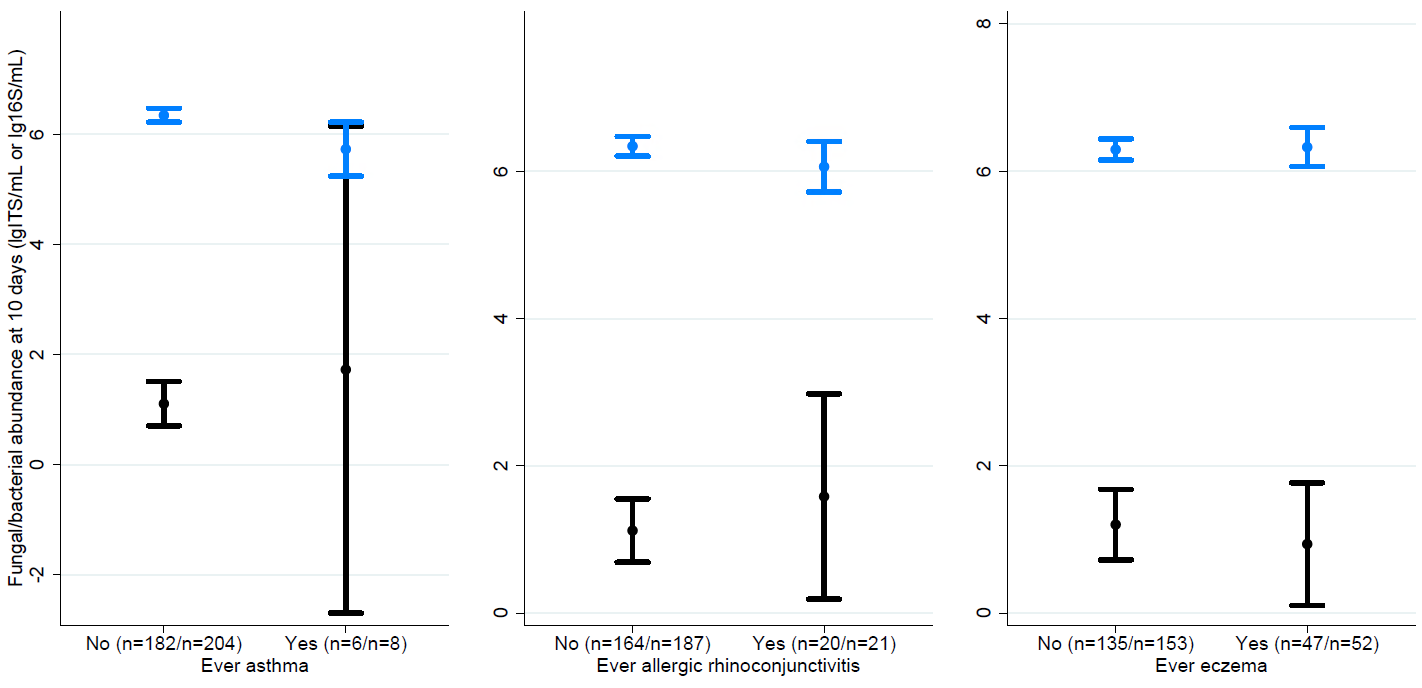

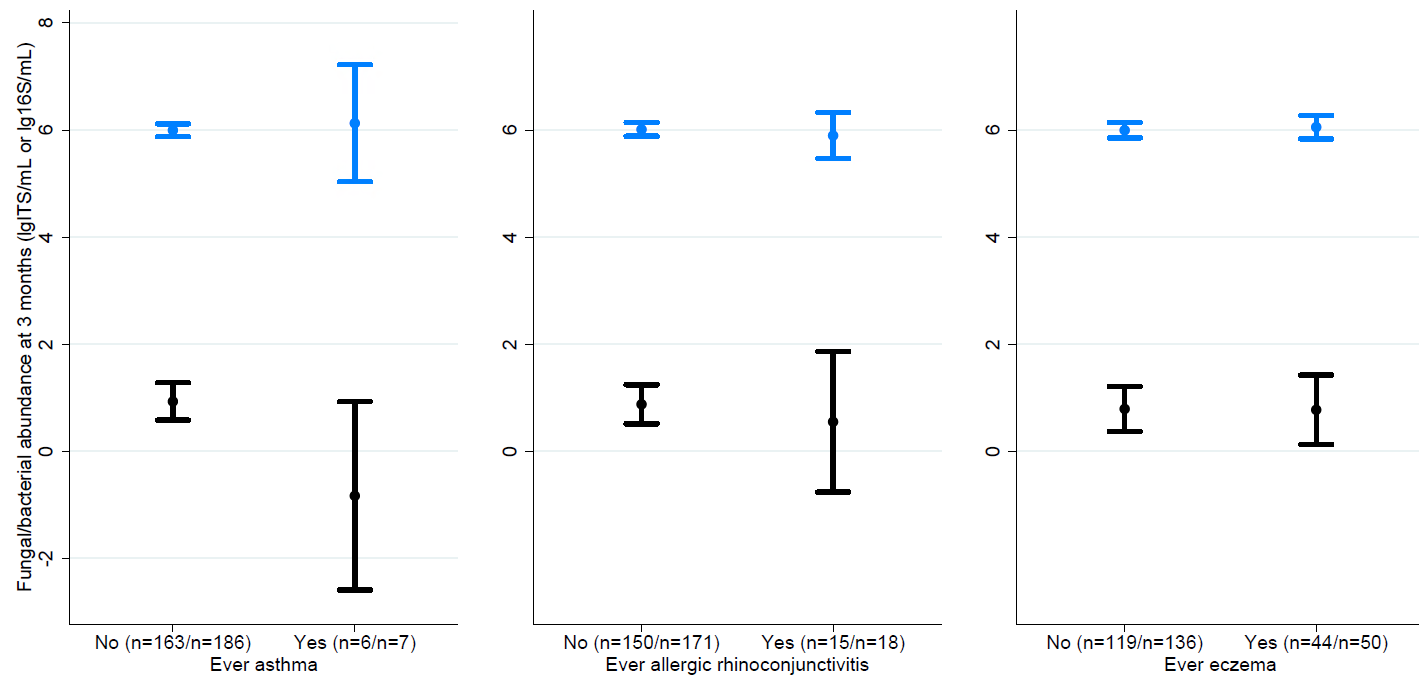
B**

**
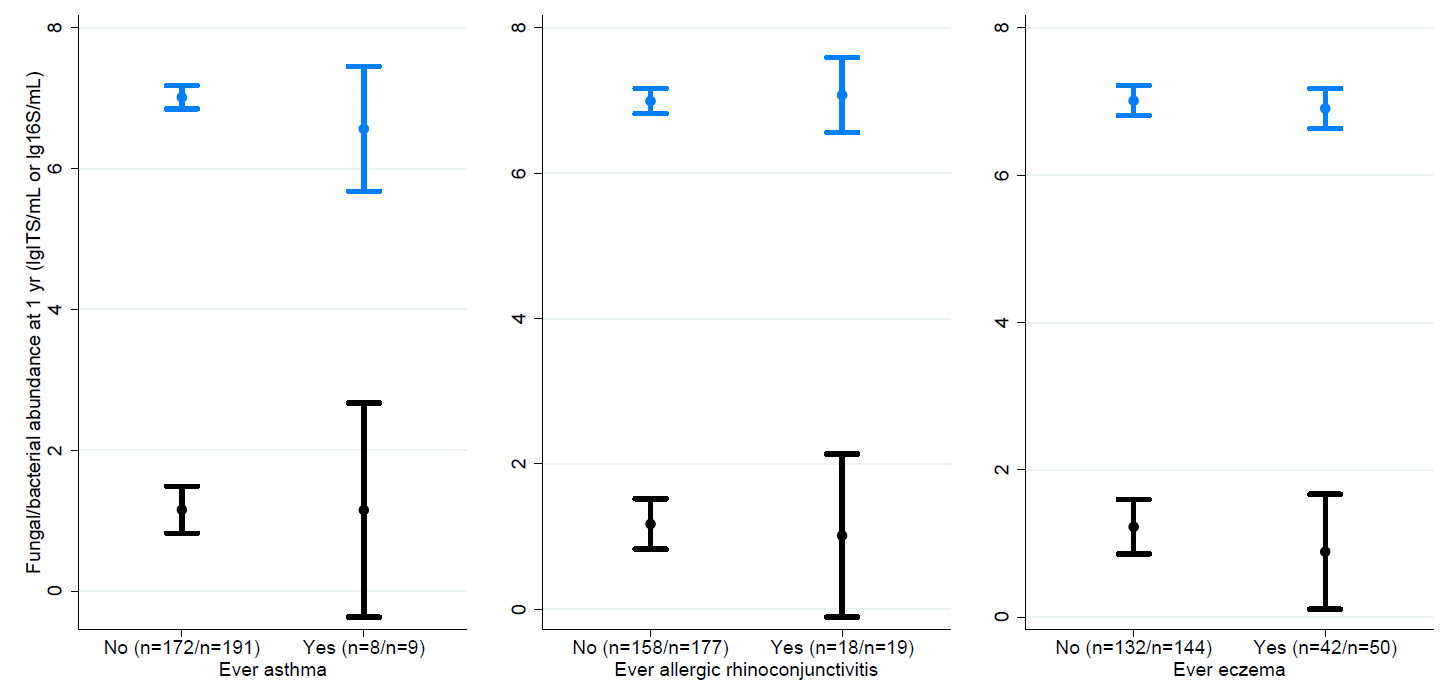
C**

**Supplementary Figure 1: Fungal and bacterial abundance and allergy-related diseases at 10 days, 3 months and 1 year.** These figures depict the mean and 95 % CI of fungal (black) and bacterial (blue) abundance at 10 days (A), 3 months (B) and 1 year of age for children with ever allergy-related disease at 0-6 years, supplementing the logistics regression analyses. The numbers within brackets represent the number of participants in each group for fungal and bacterial analysis, respectively.

**
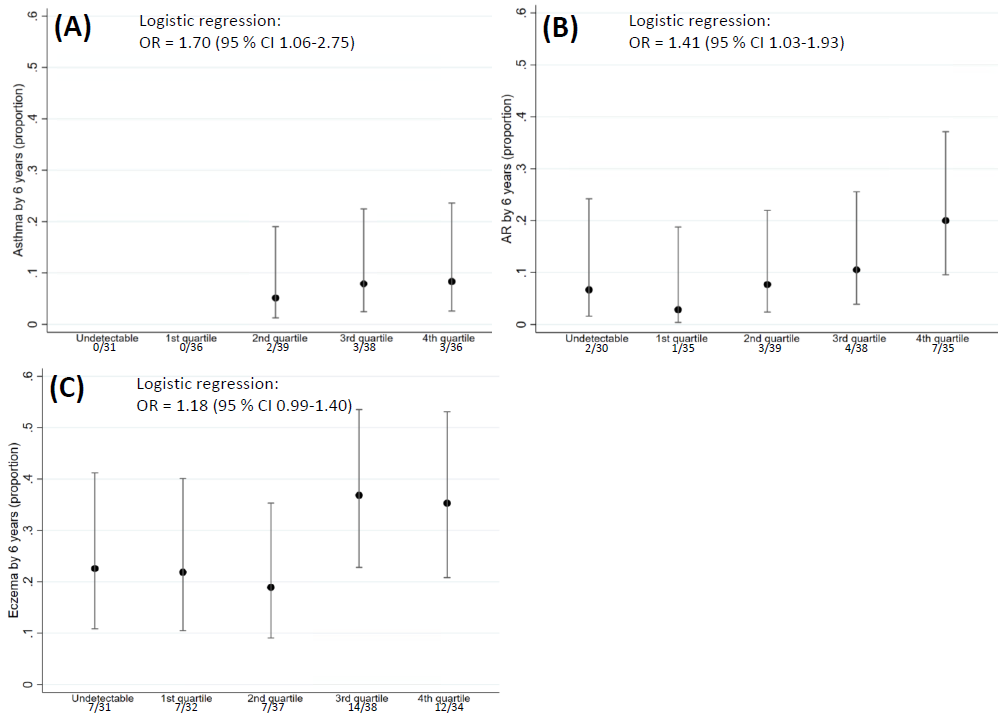
**

**Supplementary Figure 2:** Observed proportion of children with ever (A) asthma, (B) allergic rhinoconjunctivitis and (C) eczema by 6 years of age depending on the fungal abundance was undetectable or fell within the first, second, third or fourth quartile of fungal abundance among those with detectable levels of fungus. The number of children with each of the allergy-related diseases and the total number of children within each group are displayed below each category. The odds ratio (OR) and 95 % confidence interval (CI) displayed on each graph is from logistic regression with allergy-related diseases as the dependent variable and included as a log-transformed continuous independent variable.

**
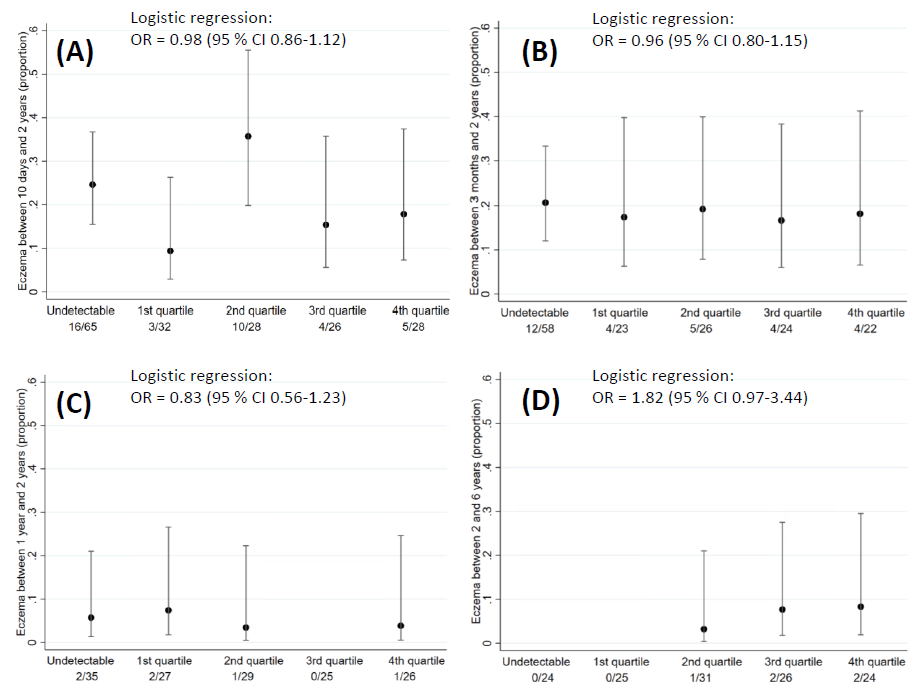
**

**Supplementary Figure 3.** Observed proportion of children who developed eczema subsequently to each timepoint of stool collection and based on the fungal abundance in their stools at the respective timepoints. Here fungal abundance is categorised into 5 categories: Undetected, and the first, second, third and fourth quartile for those with detectable abundances. (A) Illustrates the proportion of children who developed eczema between 10 day and 2 years of age based on fungal abundance at 10 days; (B) proportion of children who developed eczema between 3 months and 2 years by their fungal abundance at 3 months; (C) proportion of children who developed eczema between 1 year and 2 years by their fungal abundance at 3 months and (D) the proportion of children who developed eczema between 2 and 6 years by their fungal abundance at 2 years. The number of children with eczema and the total number of children within each group are displayed below each category. The odds ratio (OR) and 95 % confidence interval (CI) displayed on each graph is from logistic regression with eczema as the dependent variable and included as a log-transformed continuous independent variable.

**
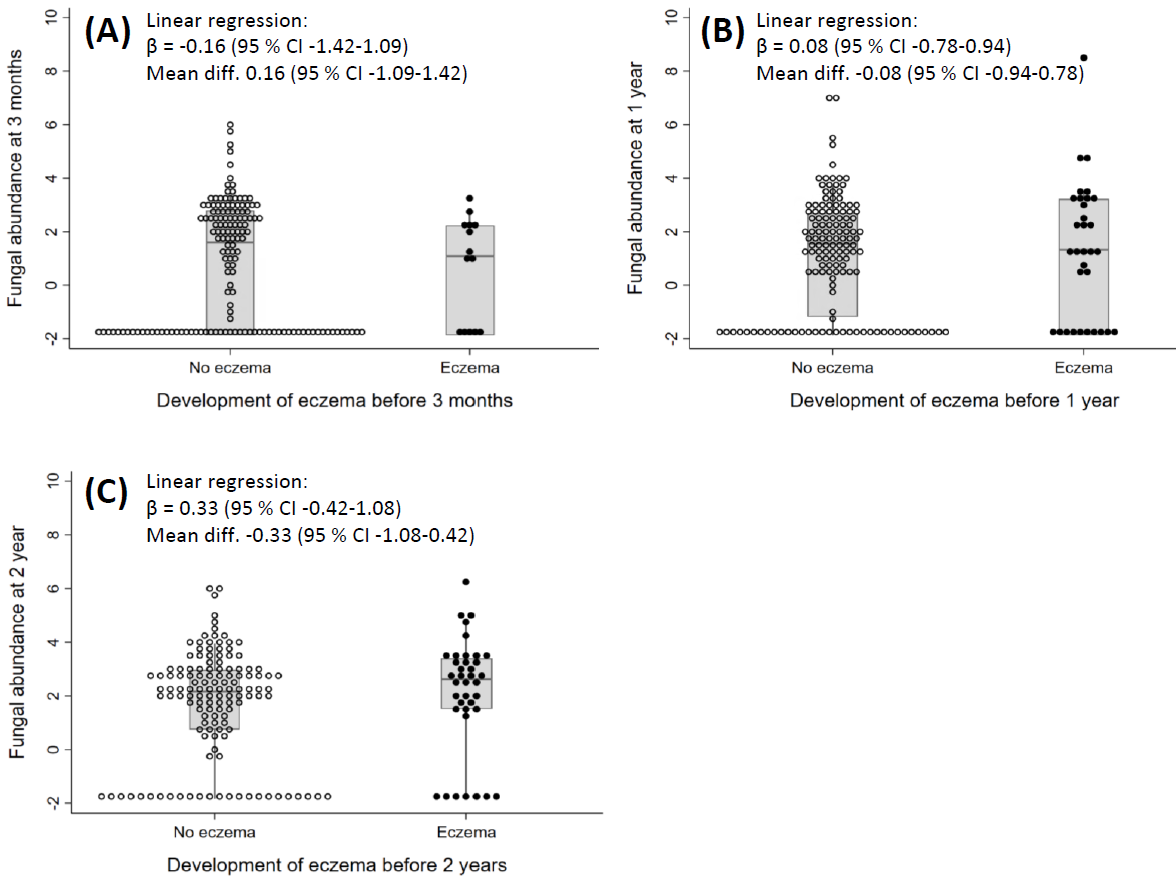
**

**Supplementary Figure 4.** Observed log-transformed fungal abundances at (A) 3 months, (B) 1 year and (C) 2 years depending on whether the child had developed eczema prior to the respective stool collection timepoint. The mean difference between the groups and 95 % confidence interval were estimated using linear regression and are reported on each graph. Among children with available stool samples at 10 days, only 1 had reportedly developed eczema prior to 10 days and this timepoint is therefore omitted.


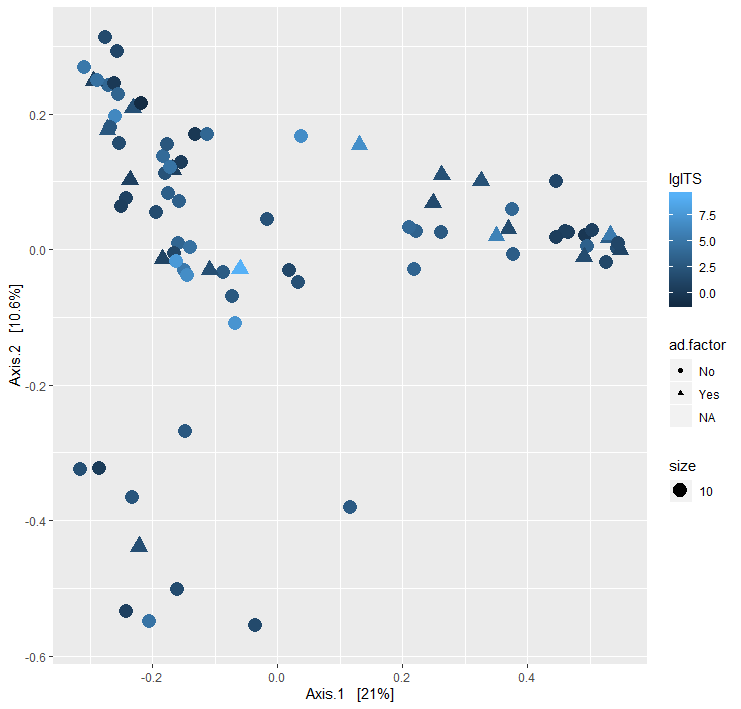


A


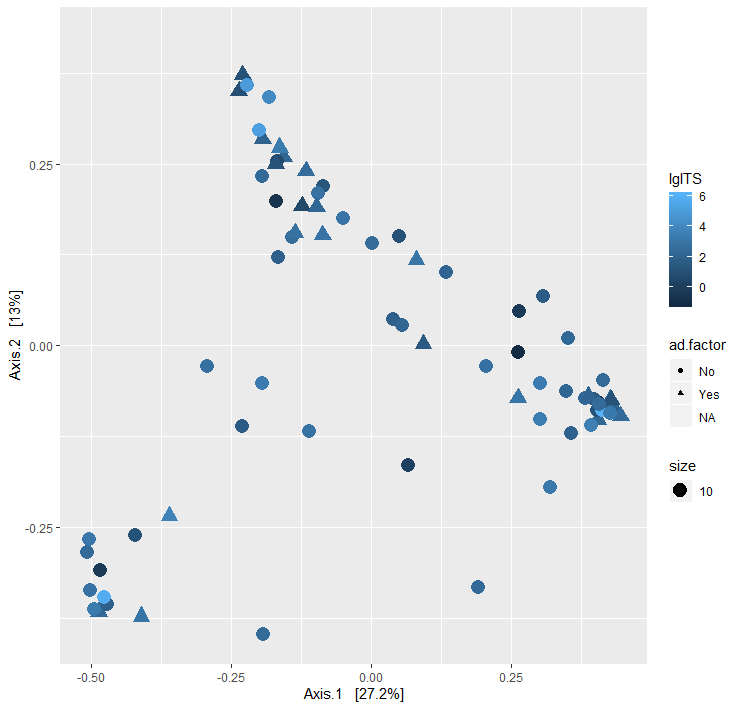


B


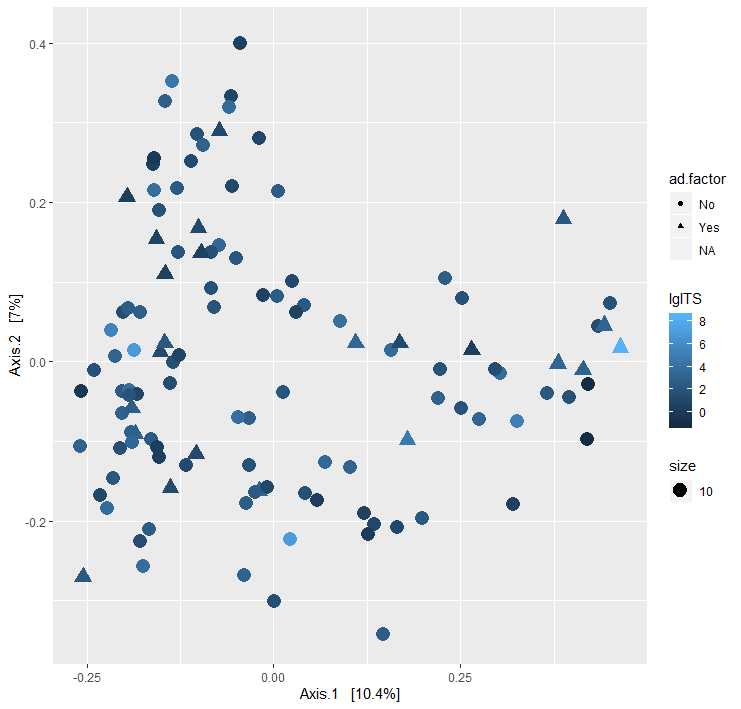


C


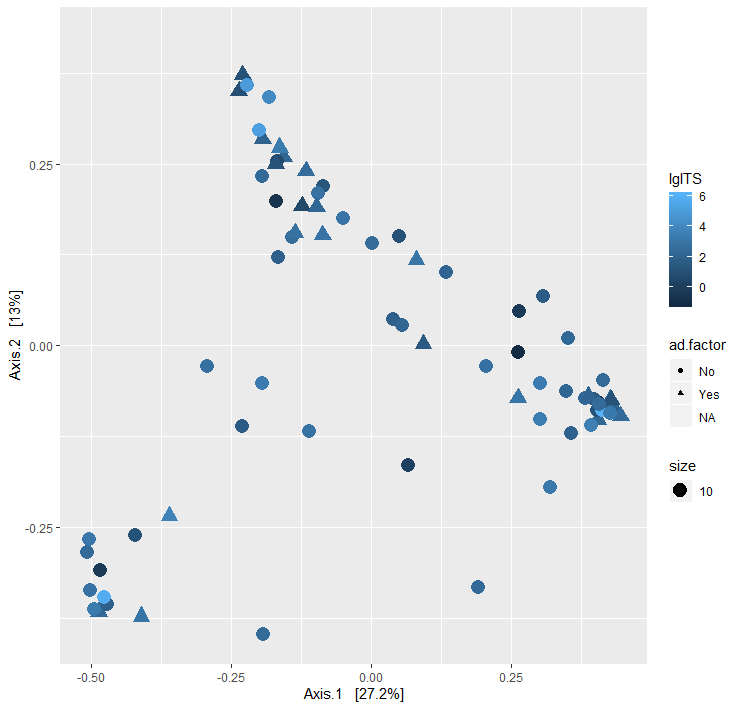


▲Ever AE
 ● Not ever AE

D


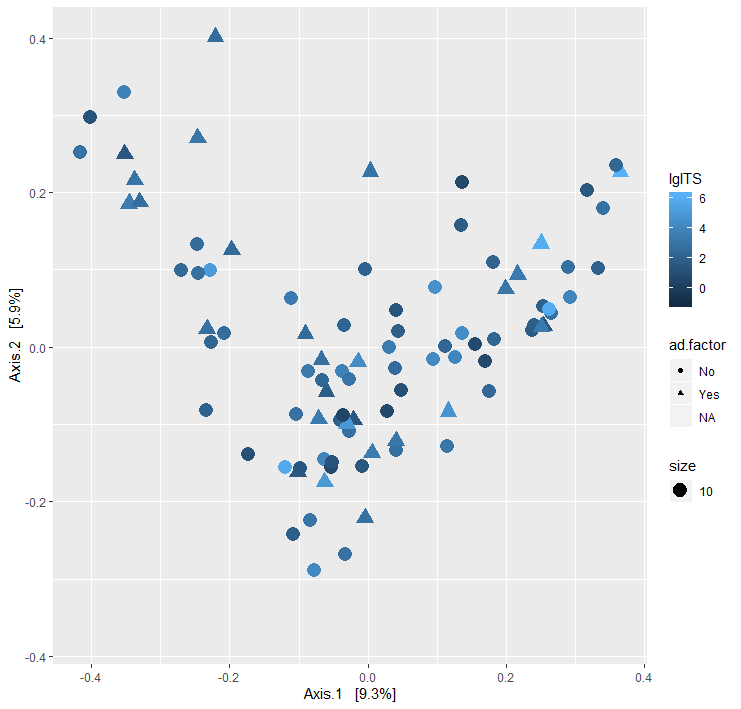
**Supplementary Figure 5. PCoA plot for age groups.** Individually ordinated PCoA plots for 10-day (A), 3-month (B), 1-year (C) and 2-year (D) samples, ligher blue colour for increasing fungal abundance (lgITS), triangles as ever AE and circles as not ever AE.

D


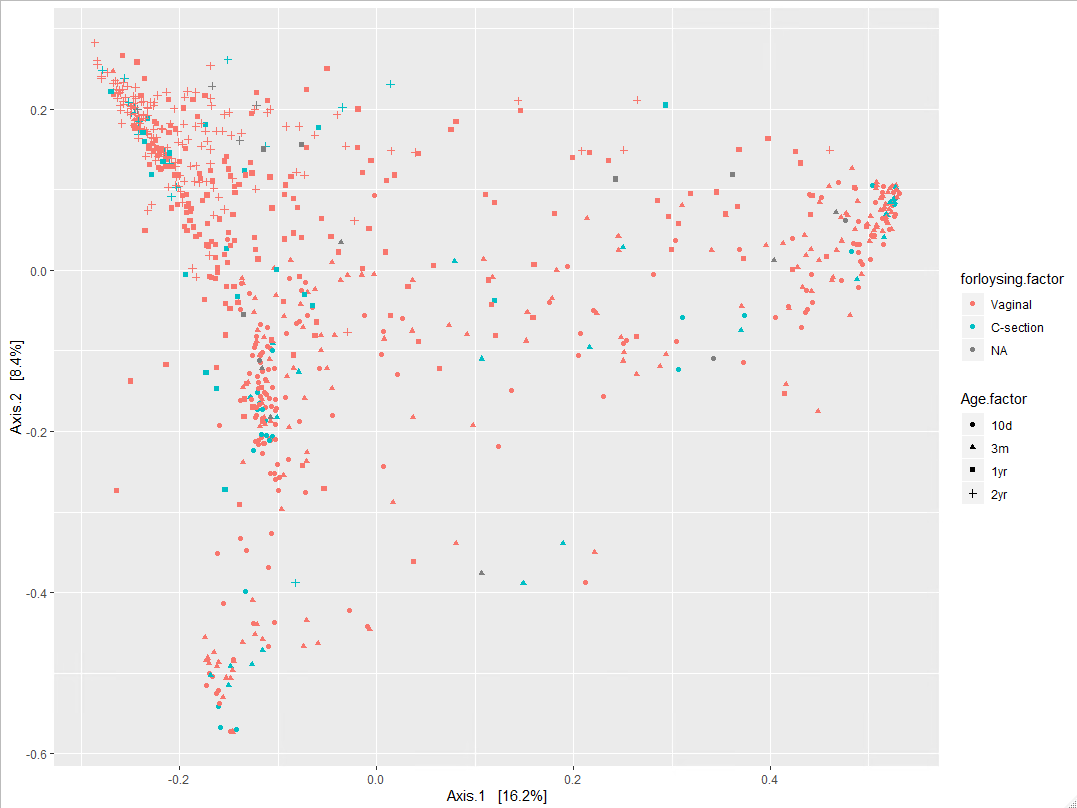


▲3 months
● 10 days
■ 1 year
**+** 2 years
● Vaginal
● C-section

**Supplementary Figure 6A. PCoA plot of bacterial microbiota for mode of delivery and age.**


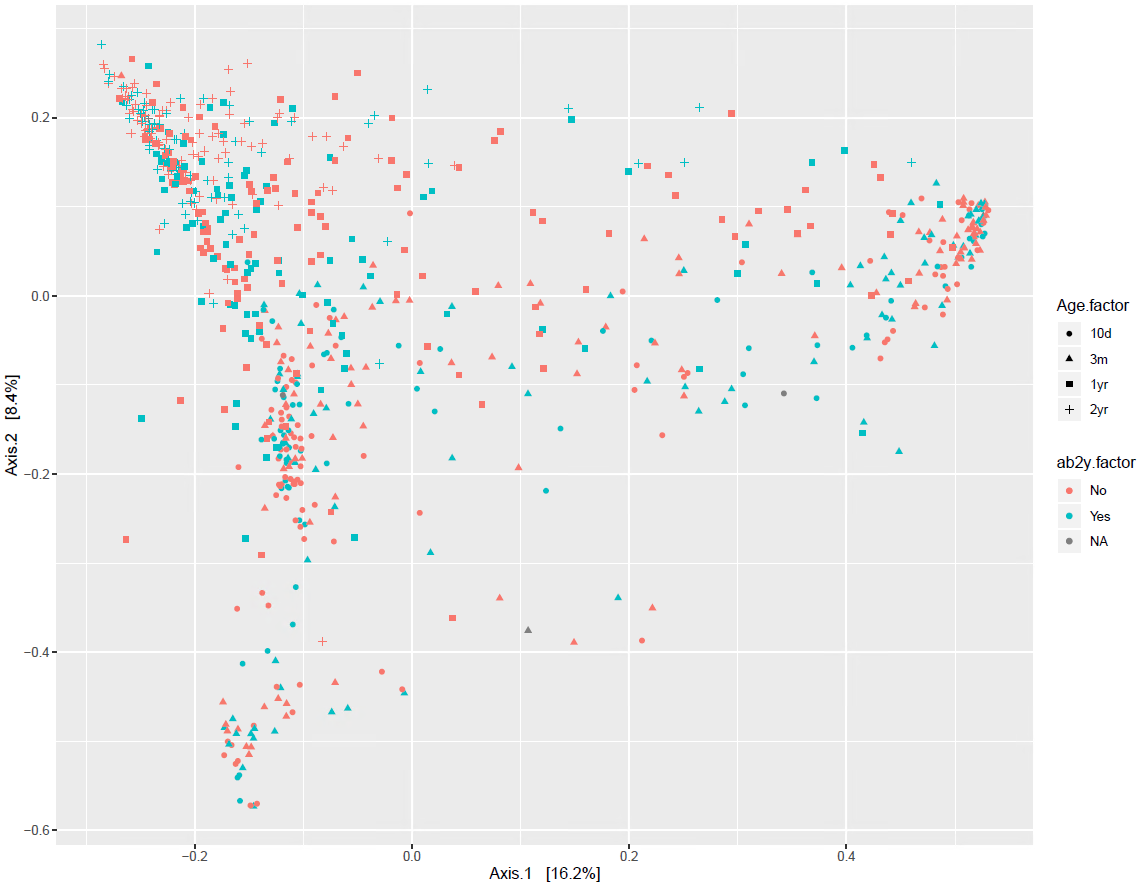


▲3 months
● 10 days
■ 1 year
**+** 2 years
● No AB at 2 years
● AB at 2 years

**Supplementary Figure 6B. PCoA plot of bacterial microbiota for antibiotic administration within 2 years and age.**

**Supplementary Figure 6C. PCoA plot of bacterial microbiota for gestational age at birth and age.**


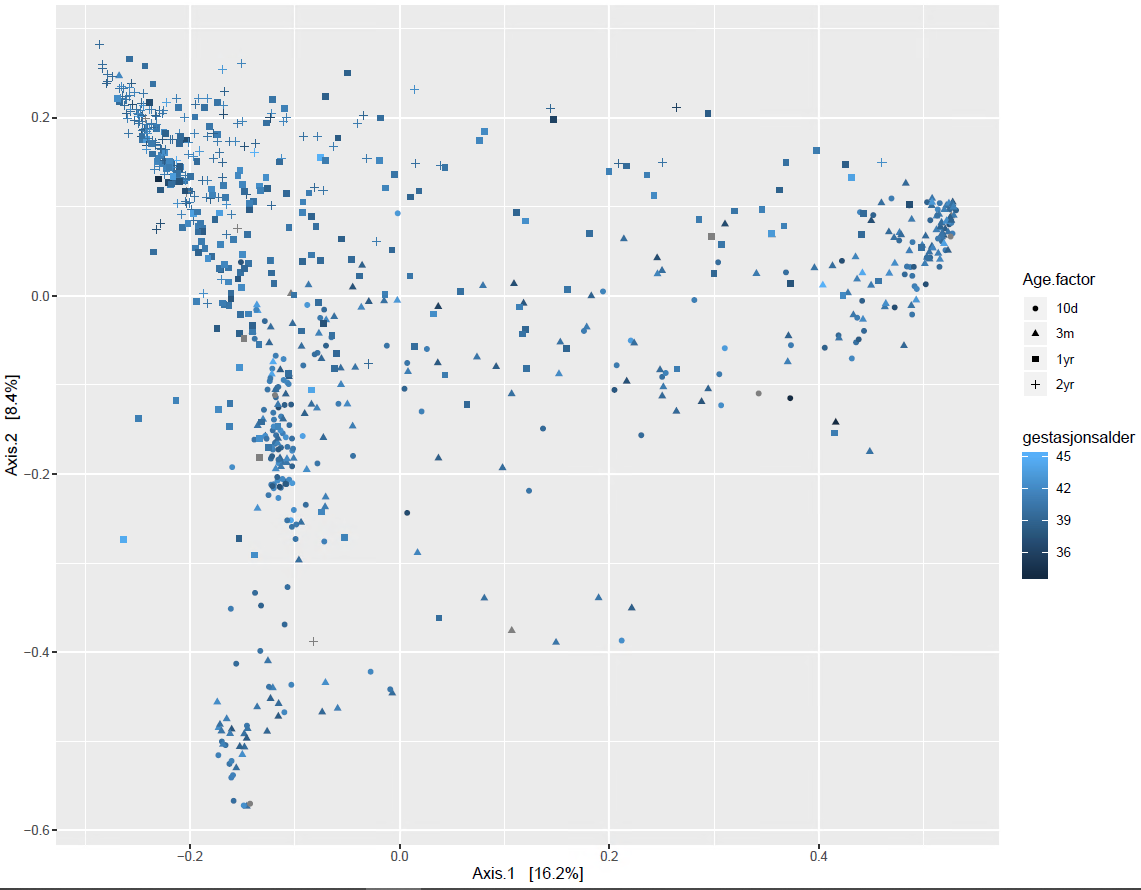


▲3 months
● 10 days
■ 1 year
**+** 2 years

Gestational age


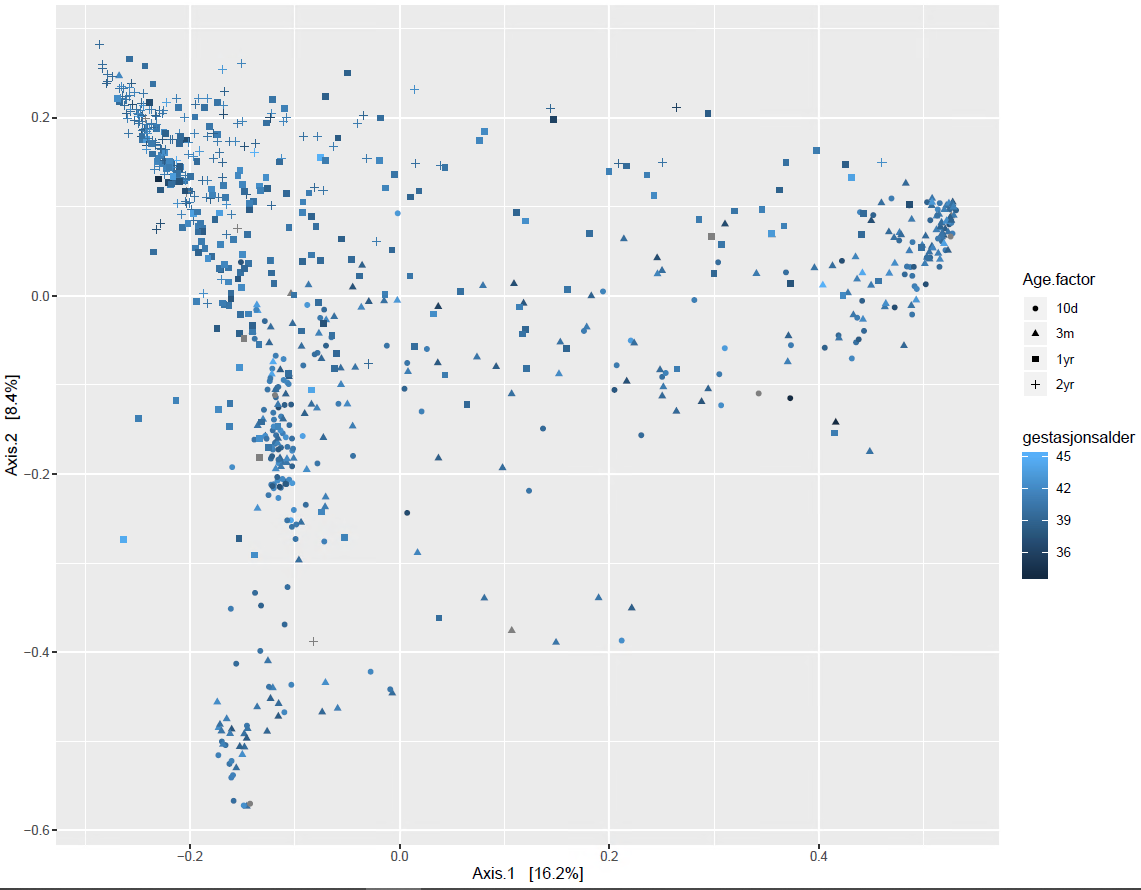


**Supplementary Figure 6D. PCoA plot of bacterial microbiota for age at weaning (months) and age.**


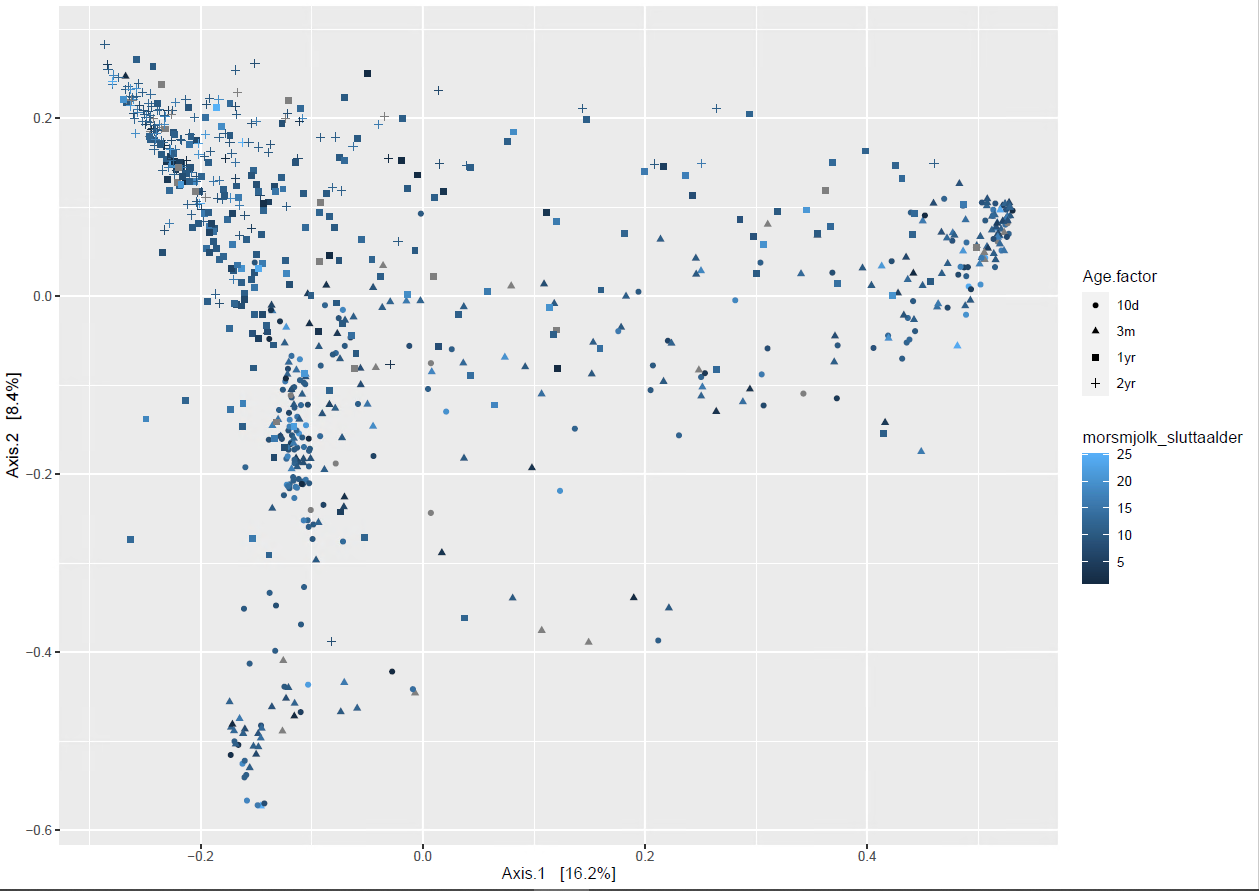


▲3 months
● 10 days
■ 1 year
**+** 2 years


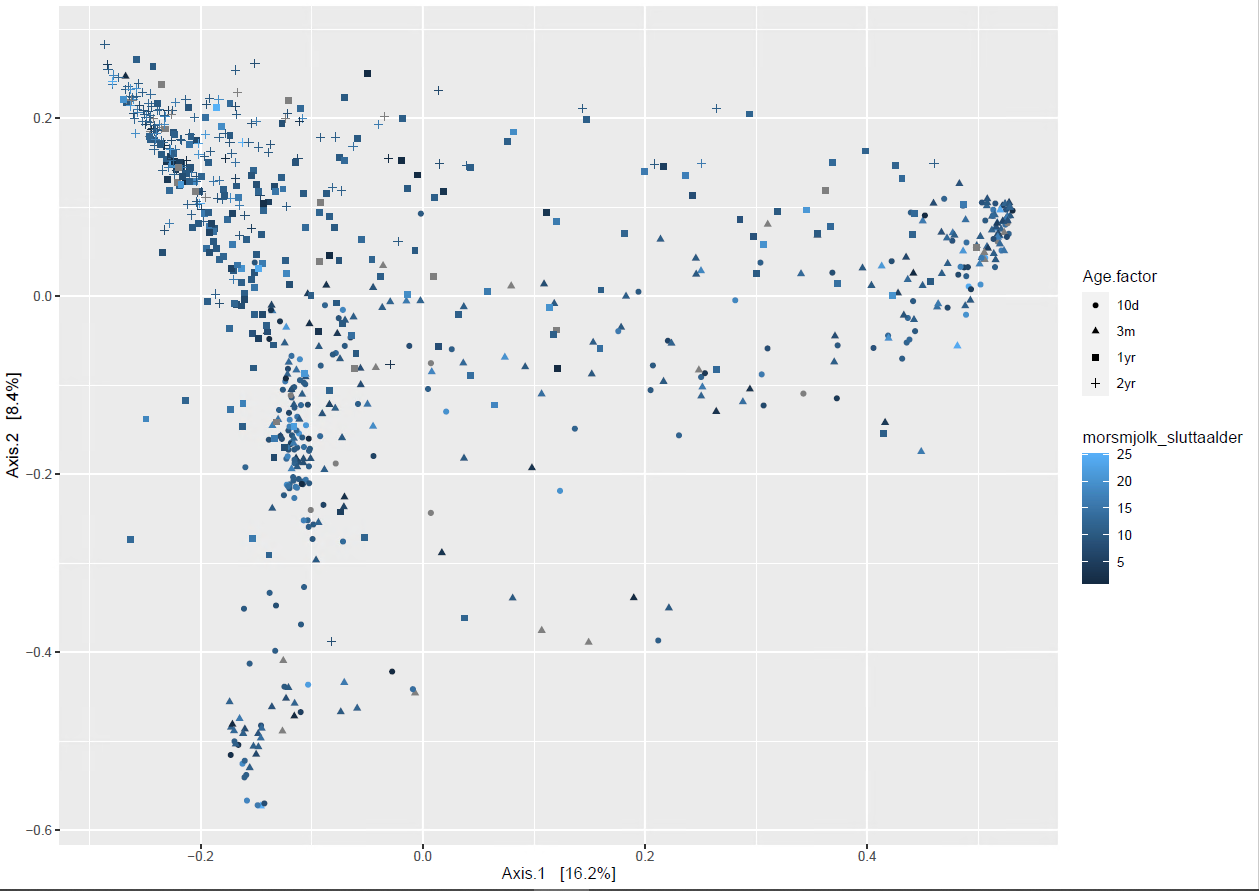


Age (mo) at weaning


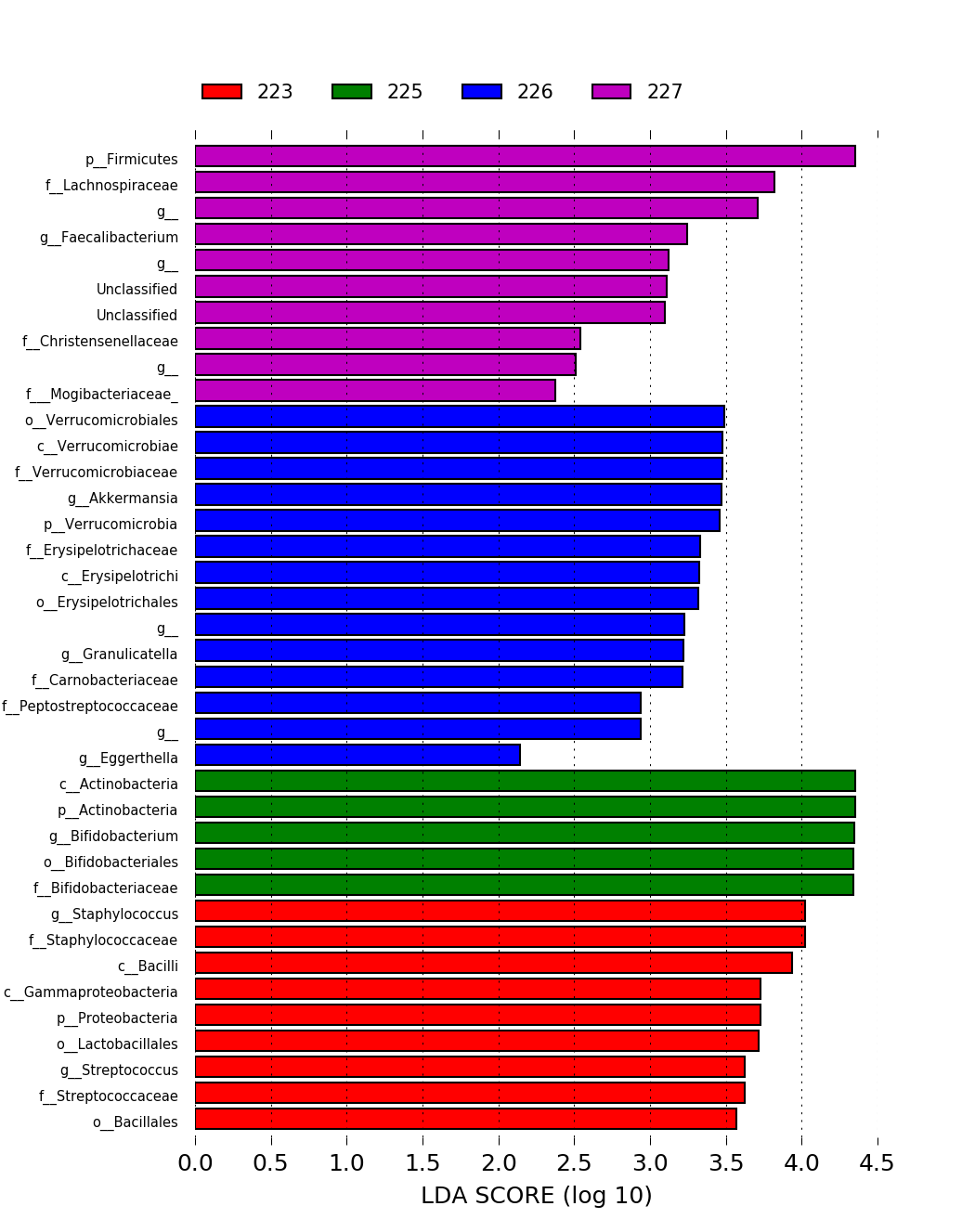


**Supplementary Figure 7A. Linear discriminant analysis effect size (LEfSe) plot for age.** LEfSe analysis for 10-day (red), 3-month (green), 1-year (blue) and 2-year (purple) sample, showing which bacterial samples are overrepresented in the different samples. Aerobic bacteria like staphylococci, streptococci and lactobacilli are overrepresented at 10 days, following a gradual shift towards bifidobacterial representation at 3 months before moving towards an adult bacterial microbiota at 1 and 2 years.


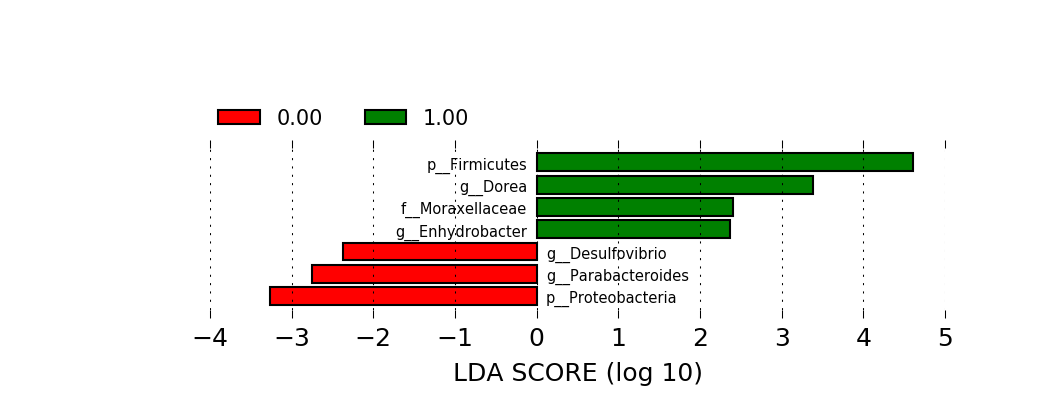


**Supplementary Figure 7B.** **Linear discriminant analysis effect size (LEfSe) plot for fungal abundance.** Children with low fungal abundance at 2 years of age (0, red) were overrepresented with Proteobacteria, *Parabacteroides* and *Desulfovibrio*, whereas those with higher fungal abundances (1, green) had a greater abundance of other Firmicutes, Moraxellaceae and *Enhydrobacter*.

1. Schei K, Simpson MR, Avershina E, et al. Early Gut Fungal and Bacterial Microbiota and Childhood Growth. *Frontiers in pediatrics*. 2020;8:572538. doi:10.3389/fped.2020.572538
